# Electromyographic responses during time get up and go test in water (wTUG)

**DOI:** 10.1186/2193-1801-2-217

**Published:** 2013-05-10

**Authors:** Antonio I Cuesta-Vargas, Carlos Cano-Herrera, Danielle Formosa, Brendan Burkett

**Affiliations:** Department of Physiotherapy, University of Malaga, Av de Martiricos s/n, Malaga, 29071 Spain; School of Clinical Science, Faculty of Health Science, Queensland University Technology, Queensland, Australia; Centre for Healthy Activities, Sport and Exercise at the University of Sunshine Coast, Sunshine Coast, Australia

**Keywords:** EMG, Aquatic, Time to up and go, Hydrotherapy

## Abstract

The aim of this study was to use sEMG to measure the neuromuscular activity during the TUG task in water, and compare this with the responses for the same task on land. Ten healthy subjects [5 males and 5 females [mean ± SD]: age, 22.0 ± 3.1 yr; body mass, 63.9 ± 17.2 kg. A telemetry EMG system was used on the following muscles on the right side of the body: the quadriceps – rectus femoris [RF], long head of the biceps femoris [BF], tibialis anterior [TA], gastrocnemius medialis [GM], soleus [SOL], rectus abdominis [RA] and erector spinae [ES]. Each subject performed the TUG test three times with five minutes recover between trials in water and on dry land. The % MVC was significantly different (p < 0.05) for majority of the muscles tested during the TUG water compared to dry land. % MVC of RF [p = 0.003, t = 4.07]; BF [p = 0.000, t = 6.8]; TA [p = 0.005, t = 5.9]; and SOL [p = 0.048, t = 1.98]; RA [p = 0.007, t = 3.45]; and ES [p = 0.004, t = 3.78]. The muscle activation of the trunk and the lower limb [VM RF, BF, TA, GM and SOL] were lower in water compared to dry land, when performing a TUG test.

## Introduction

Aquatic exercise is commonly used in rehabilitation settings and the unique features of movement in water may provide an alternative option for people unable to exercise successfully on land (Batterham et al. 2011). Understanding the physics and physiological components of water therapy is crucial for effective management of various musculoskeletal, neurological and cardiopulmonary pathologies (Becker 2009). Water has unique properties include higher density, buoyancy, hydrostatic pressure, viscosity and thermodynamics (Harrison et al. (Harrison and Bulstrode 1987); (Hall et al. 1990). Each of these key components can stimulate different physiological and biomechanical responses to exercise when comparing water to dry land training (Alberton et al. 2011). Clinically, aquatic therapy programs that included closed chain exercise, such as squats, gait, step-ups and turn, have significantly enhanced patient’s mobility and functional outcomes in hip and knee osteoarthritis (Fransen et al. 2007) as well as hip and knee replacements (Rahman et al. 2009).

The frequently used *Timed Get-Up-and-Go Test (TUG)* is a clinical tool to assess mobility and risk of falling (Weiss et al. 2011); (Menz 2003); (Lamoth et al. 2011); (Beauchet 2005); (Berg 1992); (Salarian et al. 2009). The clinical relevance of the TUG test is based on the integrating of basic functional task, such as getting up and down transitions, and transitions that require balance as the patient turns or walk in straight line (Rogers 1998). These basic functional tasks are relevant to activities in daily living and are commonly associated with falls (Tinetti, 1988).

Researchers have used various methods to assess patent’s functional movement techniques, including: video analysis (Mazza et al. 2005), optoelectronic systems (Hughes et al. 1996), goniometry (Itokazu et al. 1998) and accelerometers (Goulart et al. 1999). Despite these methods being used widely in clinical studies, clinicians have only focuses on time and ignore any other deficiency of the kinematics and kinetics movement patterns. Furthermore, the total time to perform a series of complex activities were analysed without separate the movement patterns throughout the tasks. (Salarian et al. 2010); (Zampieri 2011).

Gait training and falls prevention in water is the most used program in aquatic therapy (Cuesta-Vargas 2012); HyDAT Team (2009). TUG test is one of most used instruments for assessment in the context of evidence based clinical reasoning. Changes in *muscle activity* in an aquatic environment around the trunk and lower limb have been studied using treadmill walking (Barela et al. 2006), running (Haupenthal 2010), hopping (Triplett et al. 2009) and trunk exercises (Bressel et al. 2011). The TUG test is used in aquatic programs for trunk and lower limb rehabilitation, however the neuromuscular characteristics of the TUG movement in water have not been previously described.

The surface electromyographic [sEMG] signal represents the electrical signal generated by skeletal muscles and detected over the skin surface (Merletti et al. 2009). The sEMG was highly correlated to the muscle force, however the largest disadvantage of predicting the muscle force from sEMG was that the force generated by a muscle cannot be directly measured non-invasively (Disselhorst-Klug et al. 2009), but can provide information on muscle activation and neural control strategies which are important in rehabilitation (Merletti et al. 2009). The aim of this study was to use sEMG to measure the neuromuscular activity during the TUG task in water, and compare this with the responses for the same task on land.

## Methods

### Subjects

Ten healthy subjects [5 males and 5 females [mean ± SD]: age, 22 ± 3.1 yrs; height, 172 ± 9.0 cm; body mass, 63.9 ± 17.2 kg] agreed to participate in this study. The Research Ethics Committee from the Faculty of Nursing, Physiotherapy, Podiatry and Occupational Therapy, University of Málaga [Spain] approved this study. All volunteers were explained the procedures and potential risks and written informed consent were obtained prior to data collection.

### Experimental procedures

Subjects participated in two sessions: (i) familiarization and (ii) test session. The sessions were conducted at least one hour apart.

### Familiarization session

Familiarization was conducted to orientate the subject with the protocol for the TUG test both in water and on dry land. During this session, the subject received verbal feedback from the investigators regarding their form in the TUG test.

### Timed-get-up-and-go test

Each subject performed the TUG test three times with five minutes recover between trials. All subjects used an armless chair and were instructed not to use their arms to stand up. Although in traditional TUG an armchair is used (Podsiadlo & Richardson 1991), we used an armless chair. Previous studies explored using armless chairs. Using armless chairs could reduce the variability between subjects by eliminating the choice to use or not to use the armrests to arise (Salarian et al. 2010).

TUG test was conducted on 3 meter walkway. The beginning and end of the test was determined by 2.5 centimeter green tape markings on the floor. This was shown to the subject prior to the start of the testing protocol. Subjects were instructed to sit straight and their posterior side touching the back of the chair. After the tester signalled the start of the trial, subjects rose from the chair and walked at their fastest walking speed to the end of the 3 m. Once this was reached the subject turned around and returned back to the starting chair, turned around and sat down. The subjects were instructed not to run during this protocol. The performance time was recorded using a stop-watch. No feedback was provided during the exercise to the participant and the same investigator visually determined accurate execution of each repetition. If the exercise was performed incorrectly, it was repeated. Participants began each set on the verbal command “go”.

### Test session

The EMG devices were not removed between the TUG testing trials. A telemetry EMG system was used [ME 6000, Mega Electronics Ltd, Kuopio, Finland] on the following muscles on the right side of the body: the rectus femoris [RF], long head of the biceps femoris [BF], tibialis anterior [TA], gastrocnemius medialis [GM], soleus [SOL], rectus abdominis [RA] and erector spinae [ES]. For each muscle, three disposable adhesive circular Ag – AgCl electrodes [Lessa, Barcelona, Spain] were placed on the muscle along the line of the muscle fibres. Anatomical guidelines for electrode placement were followed according to Perotto et al. (2005). The inter-electrode distance was set at 2 cm. Before electrode placement, the skin surface was shaved (if needed) and cleaned with alcohol pads to minimize skin resistance (Silvers et al. 2011). For consistency, the same investigator prepared all of the subjects. The EMG leads were connected to a transmitting unit via customized cables.

Maximum voluntary isometric contraction [MVC] tests were performed in order to estimate maximal EMG amplitude for each muscle. The MVC tests were conducted on dry land for 5 seconds (s) before the performance of the TUG test on dry land. The MVC values were used for further normalization of the EMG signal (Alberton et al. 2011). The electrode placement and tests were conducted in accordance with current recommendations for the use of surface EMG (Perotto et al. 2005). After the MVC tests the subjects completed three repetitions for the TUG test on dry land using the same starting position, chair height and instructions as per the familiarization session. The EMG system was manually triggered before the command to record 5 s of data for each set. The EMG system was then put into a waterproof cover and placed around the trunk of the subject with a rubber band. The room temperature was consistently at 24°C. The order of tests was always land-water. Subjects remained at rest at least for 15 min before starting the water procedure.

After the dry land procedure, the subject performed the same task in the water, inside a swimming pool with a depth of 1 m. The same instructions were used as per the dry land testing procedure. Ambient temperature was 33°C and the water temperature was 30°C. The transmitting unit was positioned above the water at all times during the TUG test.

### Data processing and reduction

Data were filtered post-storage and the signal processed with a low-and high-pass filters (bandwidth =20Hz, attenuation=60dB and maximum frequency=500Hz). Maximum voluntary isometric contraction (MVC) tests were performed in order to estimate maximal EMG amplitude in root mean square (RMS) for each muscle. The MVC values were used for further normalization of the EMG signal.

### Statistical analysis

SPSS v15.0 was used for all statistical computations. Descriptive statistics [mean, standard deviation, minimum and maximum] were calculated for age, height, and Body Mass Index [BMI]. Standard procedures were used to calculate means and SDs. The Kolmogorov-Smirnov test showed a normal distribution of the data (P > 0.05). Each dependent variable [RF, BF, TA, GM, SOL, RA and ES muscle activity [%MVC [%]] was analyzed using a paired t-test to compare these values between the two conditions [water and land]. For all statistical comparisons, the α level was set at 0.05.

## Results

The waterproofing appeared to successfully maintain the integrity or the sEMG recordings in all conditions (Table 1). The % MVC was significantly different (p < 0.05) for majority of the muscles tested during the TUG water compared to dry land. % MVC of RF [p = 0.003, t = 4.07]; BF [p = 0.000, t = 6.8]; TA [p = 0.005, t = 5.9]; and SOL [p = 0.048, t = 1.98]; RA [p = 0.007, t = 3.45]; and ES [p = 0.004, t = 3.78] (Table 2). The GM was higher on the dry land condition compared to in water, but not with significant differences [p = 0.823, t = 0.23].

**Table 1:** **Descriptive characteristics of the 10 subjects**

	Minimum	Maximum	Mean	Std. Deviation
Age	19	30	22.0	3.1
Height	160	187	172.8	9.0
Knee-ground distance	40	52	45.9	4.3
Weight	57.5	86.6	67.8	10.1
Body Mass Index	19.9	24.8	22.6	1.7

**Table 2:** **Paired samples test of % of maximal voluntary contraction in land and water environment**

Muscle	Mean land	SD land	Mean water	SD water	Means paired difference	SD Paired difference
**RF**	23.60	16.32	4.70	8.24	18.90	14.65
**BF**	15.70	6.78	4.70	5.14	11.0	5.12
**TA**	29.50	7.32	6.40	9.39	23.10	12.35
**GM**	24.60	7.1	21.80	34.74	2.80	38.3
**SOL**	33.30	9.27	18.80	20.84	14.50	23.05
**RA**	8.40	6.20	5.40	3.8	19.2	16.04
**ES**	25.60	17.5	6.40	2.50	3.00	2.74

RF=quadriceps – rectus femoris, BF= long head of the biceps femoris, TA=tibialis anterior , GM=gastrocnemius medialis, SO=soleus, RA=rectus abdomini, ES=erector spinae.

**Figure 1:**
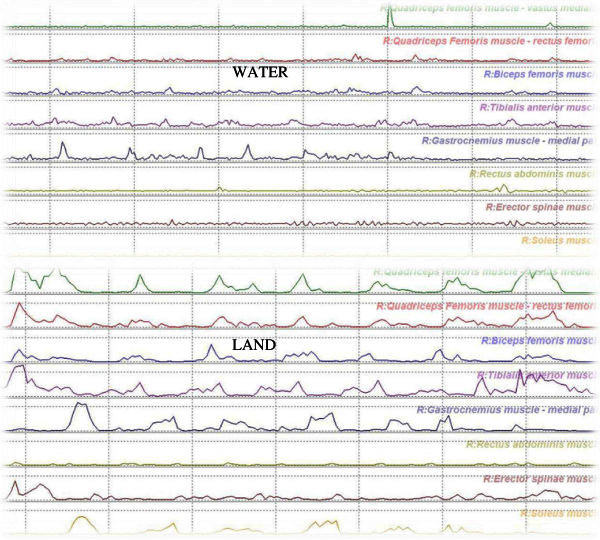
**A plot with simple EMG signals recorded in water and dry land for each muscle is shown.**

A plot with simple EMG signals recorded in water and dry land for each muscle is shown in the Figure 1.

## Discussion

The aim of this study was to measure the neuromuscular responses using EMG during the performance of a TUG test in water and dry land environments in healthy subjects. As far as the authors are aware this the first study to analyze this functional task in water. The main finding of the present study was that there were significant differences in the muscle activation of all muscles measured during the performance of the TUG task between the two environments, based differences in amplitude of EMG signals. The leg muscles activation of RF, BF, TA, SOL was significantly lower in the water. The GM was an exception and there was no significant difference between activation when comparing water to dry-land testing conditions.

The activity of the leg muscles measured in this study was lower in water than on dry land that corresponded to previous study that examined muscle activity in stance phase during walking at slower speeds (Barela et al. 2006; (Masumoto et al. 2004); (Masumoto et al. 2008); (Chevutschi et al. 2007). The differences in water and dry-land TUG muscle activation could be a result of the reduced weight bearing component of walking in the water condition. This was possibly due to buoyancy in the water condition. Immersed to the waist level resulted in off-loading and weight bearing of approximately 50% (Harrison et al. 1987). Therefore, less lower limb muscle activation in this study could be a result of less weight-bearing load in the water condition.

Researchers have identified that hydrostatic pressure when immersion in water changed the cardiovascular function, reduced lung volumes and increase breathing workload. This was a result of centra hypervolaemia (Hall et al. 1990); (Weston et al. 1987); (Choukroun et al. 1989). Furthermore, there was some interaction of the trunk muscle motor control related to posture and also the diaphragm and respiratory function (Gandevia et al. 2002). The influence of reduced lung volumes and increased work of breathing on postural stabilizing mechanisms and trunk activity with functional tasks in water is unknown. Postural responses in anti-gravity environments such as water are also not fully understood. The influence of delayed anticipatory responses (Dietz et al. 1989) and load receptor response in the legs related to extensor muscle activity (Dietz et al. 2000) may have also have some influence on the TUG test in water.

Surface EMG in water has been used in research for many years. Several studies investigated the muscle activation and identified that there was no differences in force output however there was reduced muscle activity via sEMG (Poyhonen et al. 1999); (Pinto et al. 2010); (Silver et al. 2011). A published recent tool allows a feature evaluation based on different models (e.g., linear, quadratic and exponential) allowing a better understanding of the EMG-force relationship (Andrade et al. 2012). Although there could be some minor issues related to EMG signal factors the most likely explanation is that the weightlessness or buoyancy effect on neuromuscular system is still not fully explained (Poyhonen et al. 1999).

The results presented in this study are useful in describing the functional movement of the TUG test in water to aid clinical decision-making in aquatic rehabilitation programs. Less muscle activity in the lower limb may allow people with reduced lower leg strength to successful completion of the TUG movement by controlling the movement in water. The limitation of this study was the findings were based on differences in amplitude of EMG signals only and force was not measured directly. Future studies should consider measuring force and kinematics (tridimensional displacements, linear and angular velocity,…) that may be relevant related to the TUG movement in water. Also, optimal electrode positioning could be used follow the new approach (Barbero et al. 2012), however the limitation induced by a electrode positioning can be counterbalanced by using a paired protocol. The results of this study apply only to young and healthy subjects therefore should not be generalized to a population with musculoskeletal injuries or in the elderly. Another possible limitation of this study is the lack of randomization of the TUG test performance order between water and dry land. Future research will investigate other populations and additionally functional tasks in order to provide more information to guide aquatic rehabilitation.

## Conclusions

Time up and go test are widely used in both dry land based and aquatic rehabilitation. This study was the first to describe the neuromuscular responses in healthy subjects during the performance of the TUG test in water. The muscle activation of the trunk and the lower limb [VM RF, BF, TA and SOL] were lower in water compared to dry land, when performing a TUG test.
